# Crosstalk between m6A modification and autophagy in cancer

**DOI:** 10.1186/s13578-024-01225-5

**Published:** 2024-04-04

**Authors:** Tao Chen, Liying Zheng, Peiyue Luo, Jun Zou, Wei Li, Qi Chen, Junrong Zou, Biao Qian

**Affiliations:** 1 The First Clinical College, Gannan Medical University, Ganzhou, Jiangxi China; 2 Department of Urology, The First Affiliated Hospital of Gannan Medical University, Ganzhou, Jiangxi China; 3 Department of Graduate, The First Affiliated Hospital of Gannan Medical University, Ganzhou, Jiangxi China; 4 Key Laboratory of Urology and Andrology of Ganzhou, Ganzhou, Jiangxi China

**Keywords:** Autophagy, m6A modification, Cancer, Duality, Cancer therapy

## Abstract

Autophagy is a cellular self-degradation process that plays a crucial role in maintaining metabolic functions in cells and organisms. Dysfunctional autophagy has been linked to various diseases, including cancer. In cancer, dysregulated autophagy is closely associated with the development of cancer and drug resistance, and it can have both oncogenic and oncostatic effects. Research evidence supports the connection between m6A modification and human diseases, particularly cancer. Abnormalities in m6A modification are involved in the initiation and progression of cancer by regulating the expression of oncogenes and oncostatic genes. There is an interaction between m6A modification and autophagy, both of which play significant roles in cancer. However, the molecular mechanisms underlying this relationship are still unclear. m6A modification can either directly inhibit autophagy or promote its initiation, but the complex relationship between m6A modification, autophagy, and cancer remains poorly understood. Therefore, this paper aims to review the dual role of m6A and autophagy in cancer, explore the impact of m6A modification on autophagy regulation, and discuss the crucial role of the m6A modification-autophagy axis in cancer progression and treatment resistance.

## Introduction

Cancer is one of the leading causes of death worldwide, ranking second only to cardiovascular disease. In some developed countries, cancer deaths have surpassed cardiovascular disease deaths. As cardiovascular disease rates decline in many countries, cancer is expected to become a leading cause of death [1]. Globally, there were 19.3 million new cancer cases and nearly 10 million cancer deaths in 2020, as reported by GLOBOCAN, the 2020 oncology database [2]. Furthermore, with the growing population and aging demographics, the incidence of cancer is projected to increase significantly. The occurrence of cancer places a substantial burden on both individual health and national finances. In the United States alone, the total cost of cancer screening and treatment in 2020 is estimated to reach $17.3 billion, marking a 39% increase from 2010 [3].

Research on cancer has made considerable progress over the years. However, the development of cancer is complex, and the molecular mechanisms involved are still unclear, making prevention and treatment challenging. Early surgical treatment is often the preferred choice, but many patients are already in advanced stages with distant metastases at the time of diagnosis. In advanced stages, chemotherapy is employed to prolong survival and enhance quality of life. Unfortunately, cancer patients frequently develop resistance to treatment, which ultimately becomes a major cause of death and the greatest treatment challenge [4, 5]. Consequently, it is imperative to urgently address the early diagnosis and prevention of cancer, as well as treatment resistance. Successfully tackling this issue necessitates a comprehensive understanding of the molecular mechanisms involved.

The study of autophagy has made significant progress in addressing this challenging issue. Autophagy, initially observed through the use of electron microscopy in the 1950s, has been found to be closely linked to various human diseases, including cancer [6, 7]. The association between autophagy and cancer was first reported by Liang et al. in 1999, demonstrating that Beclin-1 induces autophagy and inhibits tumorigenesis [8]. However, the precise role of autophagy in cancer remains a subject of debate. While numerous studies have suggested that autophagy has a suppressive effect on cancer, an equal number of researchers have argued that autophagy promotes cancer development and resistance to treatment. Currently, it is believed that the role of autophagy in cancer is dual and paradoxical, as it is influenced by the specific type, stage, and genetic characteristics of the cancer cells [9].

The initiation of autophagy requires the involvement of various Autophagy-related proteins (ATGs) and regulatory proteins. For normal cells, protein synthesis and activation occur in a controlled manner, with regulation at the genetic or epigenetic level. This suggests that autophagy is genetically and epigenetically regulated. Genetic mechanisms are usually irreversible, while epigenetic mechanisms are mostly reversible [10]. Epigenetic inheritance refers to heritable changes that alter expression without changing the gene sequence or chromosome structure. Examples of epigenetic inheritance include DNA methylation, histone modification, expression of noncoding RNAs and RNA modification. Several studies have demonstrated the important role of epigenetic inheritance in autophagy [11–13]. Recent reports have shown that aberrant RNA modifications lead to dysregulation of autophagy and impact tumorigenesis. Among the various RNA modifications in eukaryotic cells, N6-methyladenosine (m6A) is the most abundant. This modification is closely associated with cancer and the regulation of autophagy. However, its specific role in the regulation of cancer autophagy remains unknown [14]. This knowledge gap motivated us to investigate the intrinsic association between m6A modification and autophagy regulation in cancer. In this review, we discuss the normal physiological functions and mechanisms of autophagy and m6A modification, as well as their roles in cancer. Additionally, we explored the role of m6A modification-mediated autophagy regulation in cancer progression and drug resistance and its potential as a therapeutic target.

## Overview of autophagy and m6A modification

**Fig. 1:**
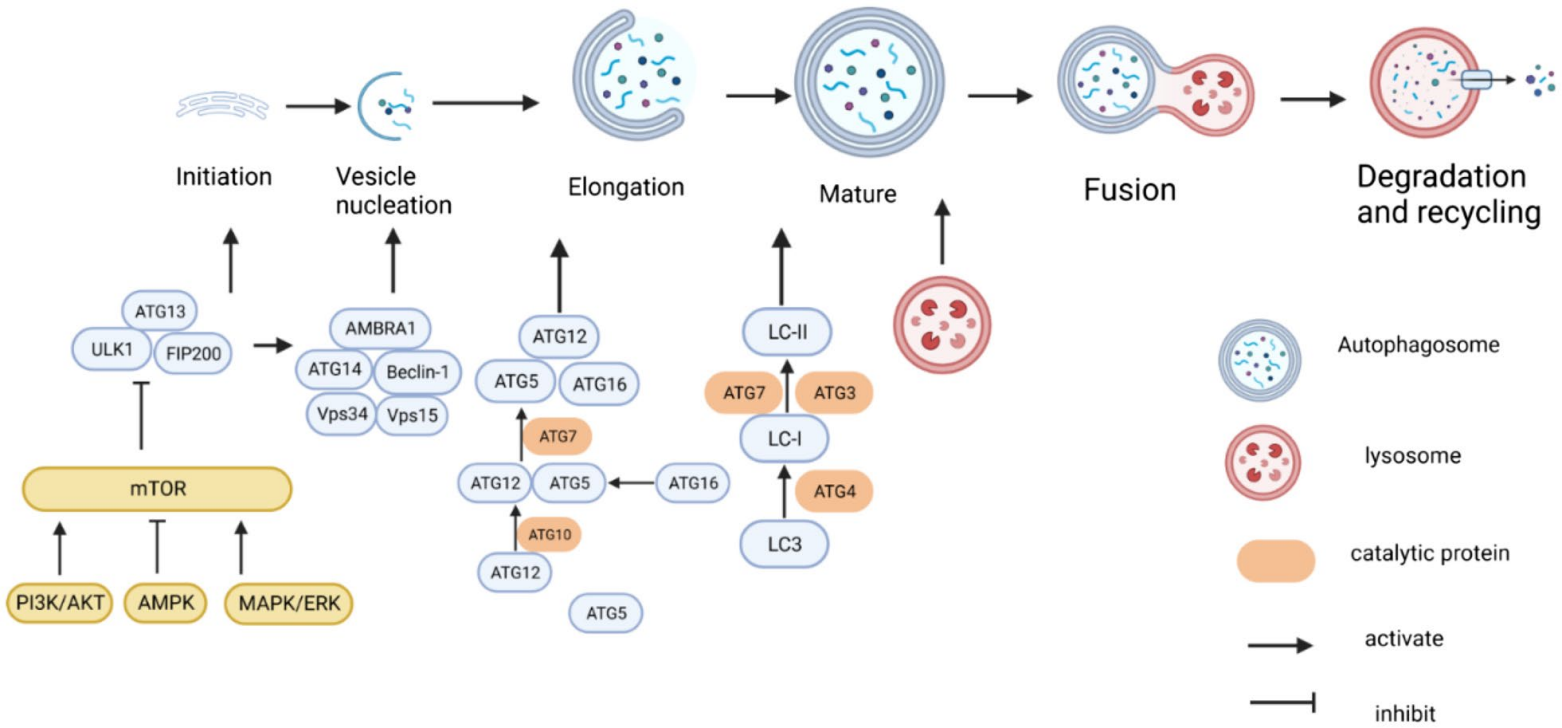
Molecular Mechanism of Autophagy With the participation of various autophagy-related proteins, autophagosomes are formed through the processes of autophagy initiation, nucleation, and elongation. Subsequently, autophagosomes fuse with lysosomes to degrade the cargoes within them. The degraded products are then recycled, providing nutrients and energy

Autophagy, also known as type II programmed cell death, is a self-digestive process in which cells use lysosomes to degrade damaged, denatured, or senescent macromolecules and organelles under the influence of external environmental factors. It encompasses three forms: macroautophagy, microautophagy, and molecular chaperone-mediated autophagy [15–19]. Understanding the molecular mechanisms of autophagy formation will contribute to improved research design and the development of new therapeutic agents in the future [6, 20]. The autophagy process can be divided into several stages: initiation, nucleation, extension, maturation, degradation, and recycling (Fig. 1) [21, 22]. Under normal physiological conditions, cells maintain a low level of basal autophagy. However, when there are cellular nutrition and energy deficiencies, accumulation of harmful proteins, or stress, the activity of the mammalian target of rapamycin protein complex 1 (mTORC1) is inhibited. This inhibition allows the activation of Unc-51-like kinase (ULK1), which in turn promotes the binding of ATG13 to the Focal adhesion kinase family interacting protein of 200 kDa (FIP200). The ATG13-ULK1-FIP200 complex, along with other ATG proteins, promotes the formation of double-membrane autophagic vesicles and initiates the autophagy process [23–26]. The nucleation process is closely related to the formation of the PI3K-Beclin-1 complex. This complex also involves ATG12, ATG5, ATG16, and microtubule-associated protein light chain 3(LC3) [27, 28]. ATG12 is initially activated by the ubiquitin-activating enzyme E1 ATG7. It is then transported and bound to ATG5 with the help of the ubiquitin-activating enzyme E2 ATG10. Finally, it binds to ATG16 to form the ATG12-ATG5-ATG16 multibody complex. This complex is localized on the surface of the outer membrane of the preautophagosomal structure and is involved in the extension of the outer membrane of the preautophagosome [29]. Additionally, the LC3 precursor is processed by ATG4 into LC3-I. LC3-I is then covalently linked to phosphatidylethanolamine (PE) to become lipid-soluble LC3-PE (also known as LC3-II). This process is facilitated by the action of the E1-like enzyme ATG7 and the E2-like enzyme ATG3 and is involved in membrane extension [28, 30]. Once the separation membrane is closed, the double membrane vesicle structure formed is called an autophagosome [30]. Subsequently, the autophagosome crosses the microtubule skeleton and fuses with the lysosome to form an autophagic lysosome. The autophagosomes and their contents are then degraded under the action of lysosomal hydrolase. The amino acids and some proteins produced during this degradation process can provide nutrients and energy or be recycled by the cells [31]. Autophagy is regulated in various ways to maintain normal autophagy levels. Mammalian target of rapamycin (mTOR) is a key regulatory protein in the initiation of autophagy that negatively regulates autophagy. Additionally, the PI3K/AKT, MAPK/ERK, AMPK, and other signaling pathways regulate autophagy by modulating mTOR [32, 33].

**Fig. 2:**
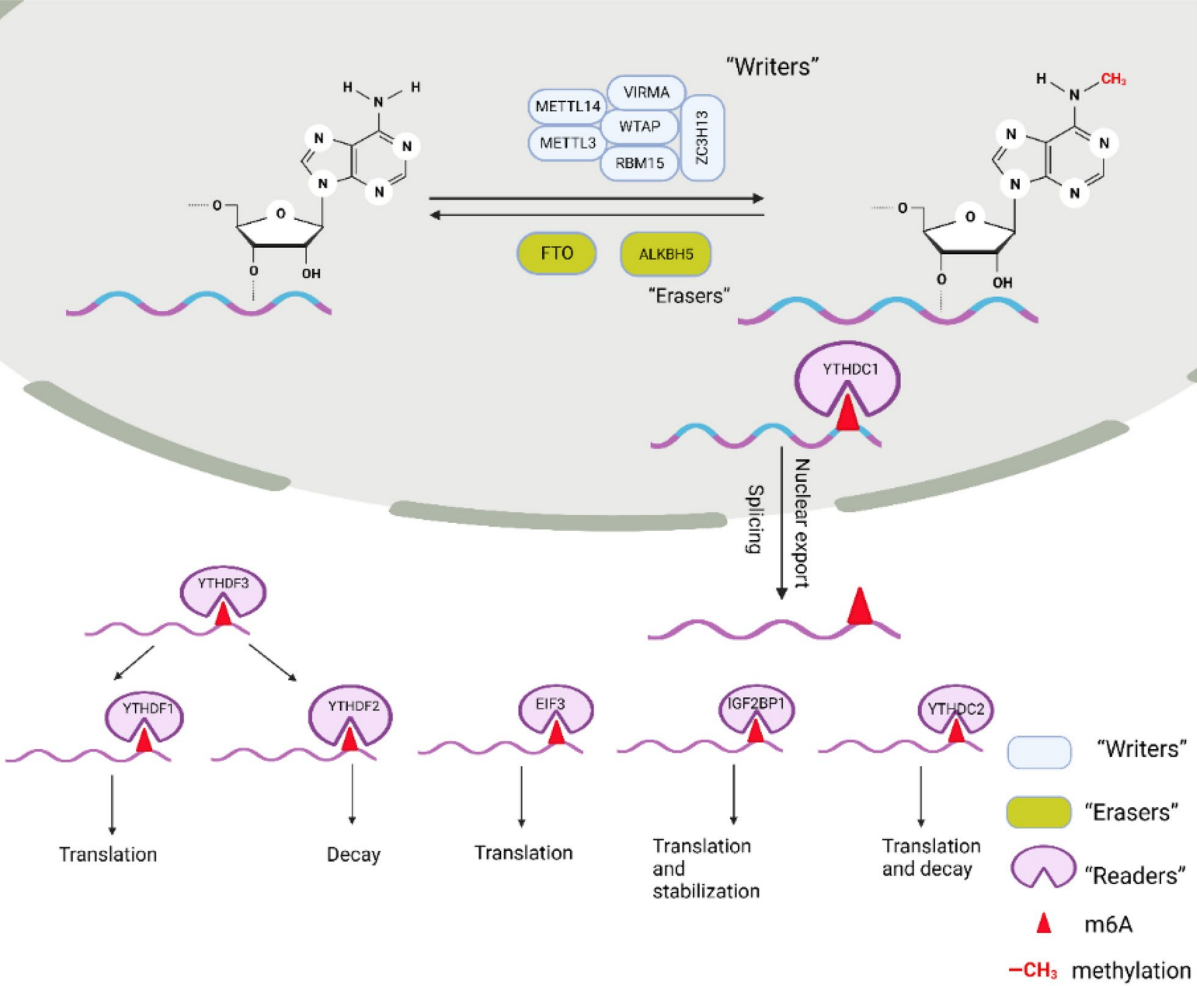
Mechanism of m6A modification. RNA undergoes m6A modification by adding a methyl group to the nitrogen atom at position 6 of adenine through the action of ‘writers’. This process is known as m6A modification and can be reversed by ‘erasers’. The m6A-modified RNA, in the presence of ‘readers’, undergoes various outcomes such as splicing, nuclear export, translation, stability enhancement, etc., which ultimately influence the expression of target genes

Currently, more than 150 posttranscriptional modifications have been identified in RNA across all organisms [34, 35]. Among these, m6A modification is the most abundant RNA modification in eukaryotic cells, with approximately 25% of cellular transcriptomes containing multiple m6A-modified residues [36, 37]. m6A modification primarily occurs in RRACH sequences (where R = A or G, H = A, C, or U), and it is predominantly enriched near the stop codon, the 3′ untranslated region (3'UTR), and long internal exons [36–39]. Furthermore, it has also been found in precursor RNAs and noncoding RNAs [40, 41]. m6A can be added by methyltransferase complexes and removed by demethylases, indicating that the process of m6A modification is dynamic and reversible in mammals [42]. Regulators of m6A can be categorized into three types: methyltransferase complexes, known as ‘writers’, catalyze the m6A modification process; demethylases, referred to as ‘erasers’, remove the m6A modification; and RNA-reading proteins, termed ‘readers’, recognize the m6A modification, bind to the RNA, and carry out the corresponding functions (Fig. 2) [43]. Methyltransferases involved in m6A modification include METTL3, METTL14, WTAP, VIRMA, RBM15, ZC3H13, and METTL16 [40, 43, 44]. Demethylases such as FTO and ALKBH5, with FTO being the first demethylase identified, have confirmed the reversibility of m6A modification [45]. ‘Readers’ recognize and bind to m6A sites, leading to different outcomes for target RNAs, such as splicing, nuclear export, translation, and degradation [42, 46]. Members of the YT521-B homology (YTH) structural domain family, including YTHDF1, YTHDF2, YTHDF3, YTHDC1, and YTHDC2, possess a conserved m6A-binding domain that selectively binds to m6A modification sites on mRNAs, resulting in diverse fates for target RNAs [36, 47]. Additionally, Insulin-like growth factor 2 mRNA-binding proteins (IGF2BPs) also bind to m6A-modified sites, enhancing mRNA stability and translation (Table 1) [48].

**Table 1:** Functions of the m6A regulator

Type	Regulator	Function	References
“Writer”	METTL3	Binds to METTL14 to form a stable heterodimer that acts as a catalytic core	[49]
	METTL14	Binds to METTL3 to form a stable heterodimer that serves as a structural support for binding to RNA	[49]
	WTAP	Ensure that the METTL3-METTL14 heterodimer is localized in the nuclear speckle	[50, 51]
	RBM15	Binds the m6A complex and recruits it to specific RNA sites	[44, 52]
	VIRMA, KIAA1429	Regulation of regioselective methylation by recruitment of MTCs	[53]
	METTL16	Catalytic m6A modification of U6-snRNA involved in pre-RNA splicing	[40]
	ZC3H13	Enhancement of m6A by ligating WTAP to the mRNA binding factor Nito	[54]
“Erasers”	FTO	Remove m6A modifier	
	ALKBH5	Remove m6A modifier	[55]
“Readers”	YTHDF1	Enhancement of m6A mRNA translation by promoting ribosome assembly and interaction with initiation factors	[56]
	YTHDF2	Selective binding and recruitment of m6A-modified mRNAs to mRNA decay sites induces transcript degradation	[57]
	YTHDF3	Interaction with YTHDF1 promotes RNA translation and interaction with YTHDF2 promotes RNA degradation	[58, 59]
	YTHDC1	Involved in RNA splicing and export	[60, 61]
	YTHDC2	Increased translation efficiency but reduced abundance of target mRNAs	[62, 63]
	IGF2BPs	Enhanced mRNA stability and translation	[48]
	EIF3	Promote mRNA translation	[64]

## Duality of autophagy and m6A modification in cancer

The exact role of autophagy in cancer regulation is still not fully understood. In their review, Klionsky et al. discussed the history of autophagy and its relationship with cancer. They highlighted that autophagy can have a dual role in cancer, either promoting or inhibiting cancer development and progression depending on the type and stage of the tumor [65]. During the early stages of tumor development, autophagy can remove misfolded proteins, damaged organelles, and ROS, providing a protective effect on the cell [66]. It helps to limit inflammatory responses, maintain genomic stability, and act as a suppressor of tumorigenesis. However, as cancer progresses, tumor cells exploit the stress-reducing property of autophagy to cope with environmental stresses and unfavorable factors, promoting tumor development [9]. For instance, in fast-growing tumors, some tumor cells are located far away from blood vessels and experience nutrient and oxygen deprivation. Autophagy aids these oxygen-deprived cells by recycling energy and helping them survive.

A variety of cellular activities in cancer development and progression, including proliferation, apoptosis, migration, invasion, angiogenesis, and drug resistance, are closely linked to autophagy. The dual nature of autophagy's effects on tumors is also reflected in its dual effects on these biological behaviors of tumor cells. For instance, pancreatic ductal adenocarcinoma and lung cancer have been found to have high levels of autophagy, which is crucial for maintaining tumor growth. Inhibition of autophagy, on the other hand, can result in tumor regression and reversal of malignancy [67, 68]. However, some examples contradict the role of autophagy in promoting tumor cell proliferation. In lymphoma and breast cancer cells, activation of autophagy has been shown to induce cell cycle arrest and inhibit the proliferation of cancer cells [69, 70]. Similarly, autophagy has dual impacts on cancer metastasis, with the ability to either enhance or reduce cancer migration and invasion under different circumstances [71, 72]. One study suggested that autophagy inhibits breast cancer metastasis by degrading the autophagy cargo receptor NRB1 [73]. However, other studies have reported that autophagy promotes cancer metastasis. In lung and liver cancers, for example, autophagy promotes epithelial-mesenchymal transition, leading to increased cancer cell metastasis and invasion [74, 75]. The roles and functions of autophagy and apoptosis are interconnected and mutually influence each other. Autophagy and apoptosis work together to promote cell death, but autophagy can also counteract the apoptotic effect and promote cell survival [22, 76–78]. A study conducted on neuroblastoma cells revealed that overexpression of ATG5 or beclin-1 reduced angiogenesis, while silencing of ATG5 and Beclin-1 had the opposite effect [79]. Similarly, in triple-negative breast cancer, the combination of herbal monomers called SANT increased autophagic flux, inhibited tumor growth, and suppressed angiogenesis [80]. On the other hand, the deletion of the Beclin-1 gene in a mouse melanoma tumor model resulted in enhanced angiogenesis [81]. These findings suggest that autophagy may limit excessive angiogenesis in tumors. However, there are also studies indicating that autophagy in tumor cells can promote angiogenesis. For example, in bladder cancer cells, autophagy induces the secretion of extracellular vesicles, increases vascular endothelial growth factor A(VEGFA)expression, and facilitates angiogenesis [82]. In breast cancer, chaperone-mediated autophagy regulates glycolysis to promote angiogenesis [83]. Finally, autophagy plays a role in modulating the sensitivity of cancer cells to chemotherapy. On the one hand, there is increasing evidence suggesting that autophagy is paradoxically activated as a protective mechanism, mediating the acquired drug-resistant phenotype of certain cancer cells during chemotherapy. On the other hand, autophagy can also induce cancer cell death during anticancer therapy. For instance, in a study involving colon cancer cells treated with 5-FU, autophagy was found to induce autophagic cell death, which was inhibited when autophagy was suppressed [84]. Additionally, the induction of autophagy effectively inhibits the growth of cisplatin-resistant uroepithelial cancer cells [85]. These findings clearly demonstrate the role of autophagy in inducing cell death during anticancer therapy. However, other studies have shown that autophagy can promote cancer drug resistance and cell survival, while inhibiting autophagy enhances the sensitivity of cancer cells to anticancer drugs [86–89]. Although the exact role of autophagy in regulating cancer treatment sensitivity is still a topic of debate, both in vivo and in vitro data tend to support the idea that autophagy promotes the resistance of cancer cells to chemotherapeutic treatments and that inhibiting autophagy can restore the sensitivity of chemotherapy-resistant cancer cells to these drugs [90].

m6A modifications have a wide range impact on RNA metabolism, influencing various processes, such as RNA expression, splicing, nuclear export, translation, and decay. Consequently, they play a crucial role in numerous physiological and pathological processes [91–94]. Increasing evidence suggests that m6A modifications are particularly significant in the initiation and progression of cancer and that abnormal m6A modifications are strongly linked to cancer development [95–98]. In cancer, m6A modifications have a dual role, acting as a tumor suppressor by inhibiting the expression of oncogenes or promoting the expression of tumor suppressor genes and acting as a tumor-initiating factor by promoting the expression of oncogenes or inhibiting the expression of tumor suppressor genes [43]. In non-small cell lung cancer (NSCLC), m6A modifications are highly enriched in RMRP, which enhances its RNA stability and promotes cell proliferation, invasion, migration, and resistance to radiotherapy in NSCLC cells [99]. Similarly, in breast cancer, the level of m6A modification of the tumor suppressor LATS1 mRNA is upregulated, resulting in the downregulation of LATS1 expression and promoting cancer development and progression [100]. These findings indicate that m6A modification plays a role in promoting cancer development and progression. However, it has also been demonstrated that m6A modification can inhibit cancer development and progression. For instance, in colon cancer, downregulation of m6A levels reduces YTHDF2-dependent mRNA degradation, leading to increased expression of its target gene, KIF26B, and facilitating colon cancer progression and metastasis [101]. Similarly, in thyroid cancer, METTL3-regulated m6A modification enhances STTEAP1 mRNA stability in a YTHDF2-dependent manner, thereby inhibiting thyroid cancer cell proliferation, migration, and invasion [102]. In addition, there is a substantial body of research supporting the dual role of m6A modifications in cancer [98, 103–105]. Previous studies have indicated that the impact of m6A on tumor progression through the regulation of target genes is influenced by three factors. First, it depends on whether the target genes are oncogenes or tumor suppressor genes. Second, the level of m6A modifications in cancers plays a crucial role. Last, the alteration of target mRNA expression and function is mediated by'readers', which can be categorized into positive-reader-role, promoting RNA expression, and negative-reader-role, inhibiting RNA expression [43].

**Fig. 3:**
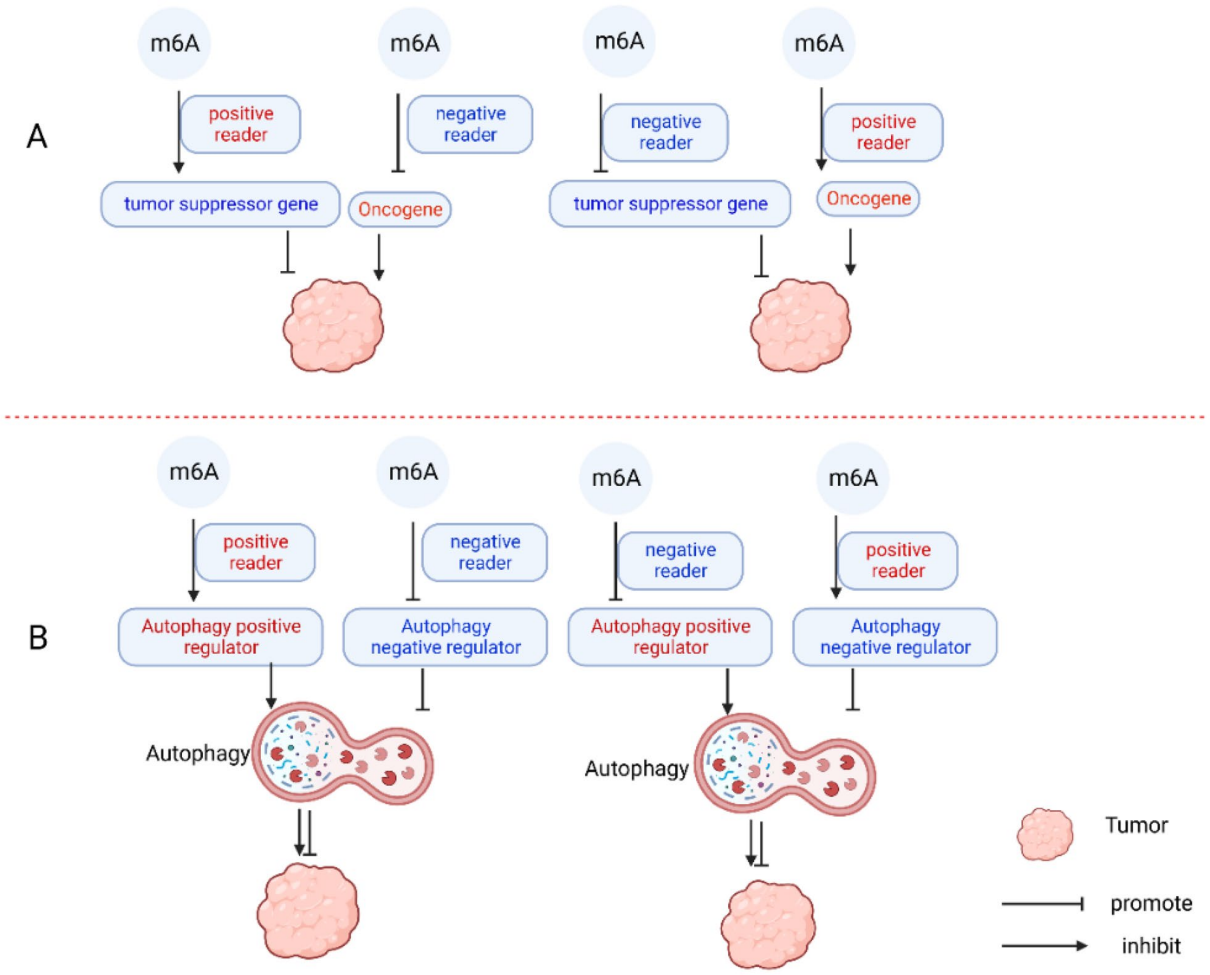
Association of m6A modification and autophagy in cancer progression

Autophagy and m6A modifications play dual roles as both tumor suppressors and tumor promoters in cancer. The role of autophagy in cancer is influenced by factors such as the tumor microenvironment, tumor type and stage, and genetic background. Consequently, the role of autophagy in cancer can vary even within the same type of cancer. Similarly, m6A modification in tumors is dependent on the type of target genes, the level of m6A modification, and changes in target mRNA expression and function mediated by'readers'. Therefore, the role of m6A modification in cancer can also differ within the same type of cancer, adding complexity and uncertainty to the roles of autophagy and m6A modification in cancer. Furthermore, autophagy and m6A modifications are interconnected, as m6A modifications target autophagy-related genes to participate in the autophagy process, thereby synergistically influencing cancer (Fig. 3). To advance the translation of research into clinical applications, it is crucial to gain a clearer understanding of the association between autophagy and m6A modifications and their respective roles in cancer.

## m6A modification-mediated autophagy regulation

Numerous studies have demonstrated the significant role of m6A modification in the regulation of autophagy. This modification can directly impact the expression of ATG genes and modulate the signal transduction mechanism of autophagy. The pioneering work by Jin et al. initially linked m6A modification to autophagy. Their study revealed that FTO specifically upregulated the abundance of the ULK1 protein, thereby facilitating autophagy initiation. Following m6A modification, ULK1 transcripts with m6A tags were targeted for degradation by YTHDF2. However, overexpression of FTO removed the m6A modification, resulting in an extended half-life of ULK1 transcripts and promoting the expression of ULK1 proteins, ultimately enhancing autophagy initiation [106]. Additionally, FTO directly targets ATG5 and ATG7, mediating their expression in a m6A-dependent manner and thus promoting autophagy. Conversely, FTO silencing leads to METTL3-mediated m6A modification of ATG5 and ATG7 transcripts. These modified transcripts are then recognized by YTHDF2 for targeting and subsequent degradation, resulting in reduced expression of ATG5 and ATG7 and inhibition of autophagy [107, 108]. Furthermore, FIP200, another crucial molecule in autophagy formation, can also undergo m6A modification. YTHDF2 recognizes and degrades m6A-modified FIP200 mRNA, thereby inhibiting autophagy. On the other hand, demethylation of FIP200 mRNA mediated by the demethylase ALKBH5 enhances autophagic flow [109]. These findings suggest that m6A modification directly impacts the expression of ATG genes, thus regulating the autophagy process. Furthermore, m6A modification can indirectly regulate autophagy by influencing the expression of autophagy regulators. For instance, in cardiomyocytes, METTL3 mediates m6A modification of TFEB mRNA, a regulator of autophagy genes, resulting in reduced TFEB expression and inhibited autophagic flow. Conversely, silencing METTL3 or overexpressing the RNA demethylase ALKBH5 has the opposite effect [110]. mTOR, an important regulator of autophagy, is also a downstream target of the PI3K/AKT and AMPK pathways. Studies have revealed that m6A modification can regulate the PI3K/AKT and AMPK pathways, suggesting that m6A modification can modulate autophagy by influencing these pathways. Specifically, M6A modification promotes the translation of PPM1A, a negative regulator of AMPK, while inhibiting the translation of CAMKK2, a positive regulator of AMPK. Consequently, m6A modification leads to reduced AMPK activity and subsequent inhibition of autophagy [111]. However, several studies have demonstrated that m6A modifications can also promote autophagy initiation. For instance, in non-small cell lung cancer, METTL3 positively regulates autophagy by increasing the expression of ATG5 and ATG7 [112]. In sensory hair cells, overexpression of YTHDF1 enhances the translation of the autophagy-related gene ATG14, thereby promoting autophagy. Conversely, deletion of YTHDF1 inhibits autophagy [113]. Additionally, METTL14-mediated m6A modification inhibits eIF4G1 expression, which further promotes autophagy [114]. Collectively, these findings indicate that m6A modifications have a dual role in the regulation of autophagy. First, m6A modifications target ATG genes and autophagy regulatory pathways, resulting in the negative regulation of autophagy. Second, m6A modifications stimulate the initiation of autophagy. These findings suggest that the impact of m6A modification on autophagy is influenced by the specific autophagy-related genes targeted by the modification, as well as the influence of 'readers' on the expression and function of these genes.

## The role of m6A modification-mediated autophagy in cancer

m6A modification and autophagy are two important and independent cellular processes that can individually or interactively contribute to tumor development. Numerous studies have demonstrated that m6A modifications can directly regulate the expression of autophagy-related proteins and pathways, thus influencing the autophagy process and participating in tumor development. Conducting systematic investigations on the mechanisms underlying autophagy, m6A modifications, and their interactions in cancer will be valuable for developing anticancer drugs and formulating therapeutic strategies. We provide a comprehensive overview of the role of m6A modification-mediated autophagy in various cancers. A comprehensive understanding of the interaction between m6A modification and autophagy will enhance our knowledge of their dual roles in cancer and contribute to the development of future cancer therapeutic strategies.

### m6A modification and autophagy in hepatocellular carcinoma

**Fig. 4:**
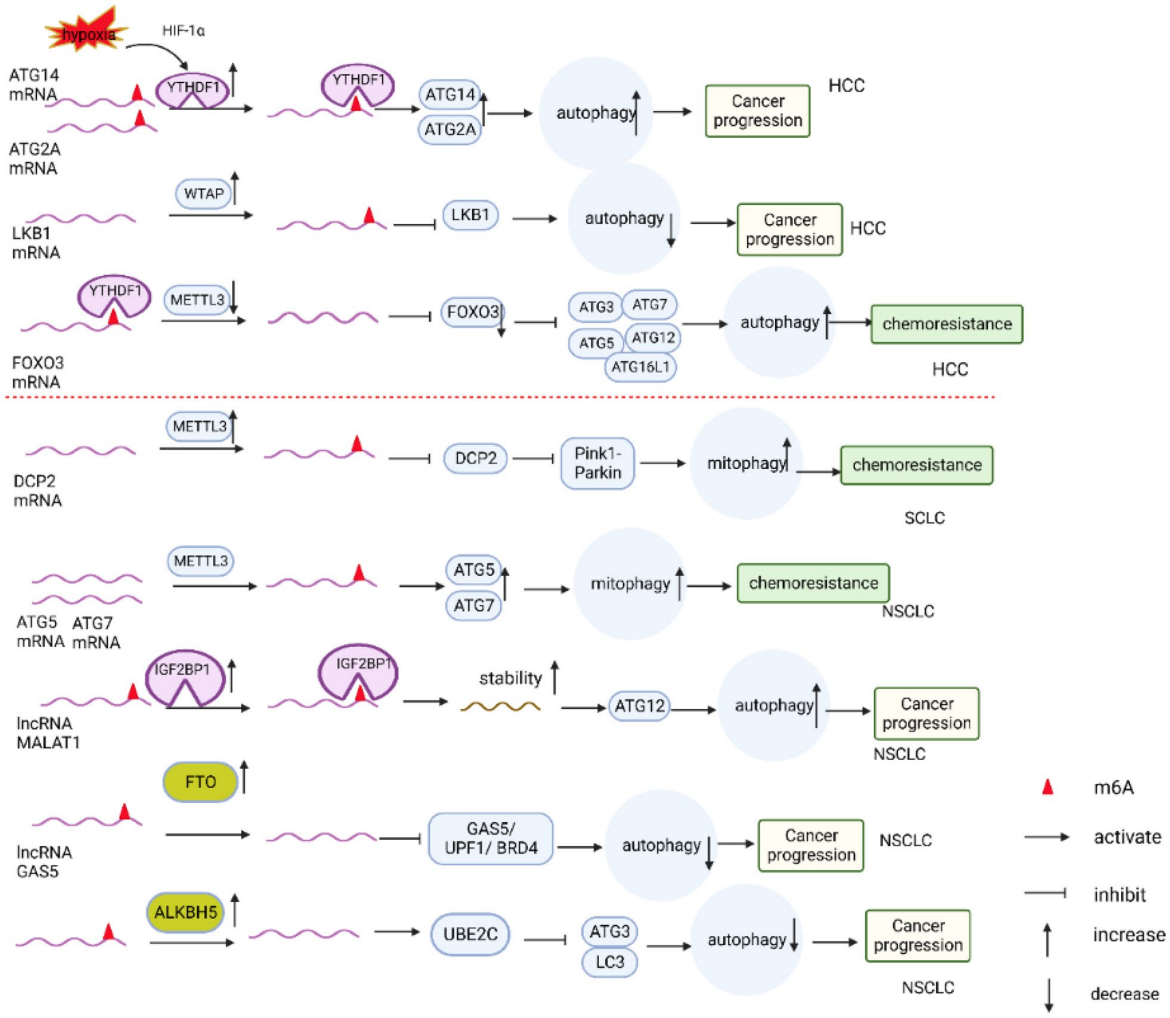
Role of m6A modification-mediated autophagy in hepatocellular carcinoma and lung cancer, m6a modification influences the expression of ATG genes or regulatory proteins that are upstream of autophagy. This regulates the levels of autophagy and impacts the progression of tumors. In the case of hepatocellular carcinoma, m6a modification affects the expression of specific target genes such as ATG14, ATG2A, LKB1, and FOXO3, thereby influencing the level of autophagy and the progression of the disease. Similarly, in lung cancer, m6a modification affects the expression of target genes like DCP2, ATG5, ATG7, and also influences the stability of LncRNA GAS5 and LncRNA MALAT1. These changes in gene expression and stability subsequently regulate autophagy and impact the progression of lung cancer

In hepatocellular carcinoma (HCC) (Fig. 4), the expression of the methylated reader protein YTHDF1 is upregulated and significantly associated with hypoxia-induced autophagy and poor patient prognosis. Silencing of YTHDF1 inhibits autophagy, as well as the proliferation, migration, and invasion ability of hepatocellular carcinoma cells. Mechanistic studies have shown that under hypoxic conditions, HIF-1α directly binds to the promoter region of the YTHDF1 gene to promote its expression. YTHDF1 promotes the translation of the autophagy-related genes ATG2A and ATG14 in a m6A-dependent manner. This facilitates the process of autophagy and the associated malignant tumor biological behaviors [115]. However, m6A modifications can also inhibit the autophagy process depending on the target gene. Enhanced WTAP expression increases the m6A modification of LKB1 mRNA, which reduces the stability and expression of the LKB1 transcript. This leads to reduced AMPK phosphorylation and autophagy inhibition and promotes the growth of HCC cells [116]. In terms of chemotherapy resistance, a study found that significant downregulation of METTL3 in sorafenib-resistant hepatocellular carcinoma activated autophagy-related pathways and promoted the expression of sorafenib-resistant and angiogenic genes [117]. The mechanism behind this was identified as METTL3 promoting m6A modification of FOXO3 mRNA, which promoted FOXO3 expression via YTHDF1. FOXO3 repressed the expression of the autophagy-associated genes ATG3, ATG5, ATG7, ATG12, ATG16L1, and MAP1LC3B. Therefore, the overexpression of FOXO3 due to m6A modification inhibited autophagy and promoted cell death in hepatocellular carcinoma cells. On the other hand, the deletion of METTL3 promoted autophagy and resistance to sorafenib by inhibiting the expression of FOXO3 [117]. FOXO3 is known as one of the first transcriptional regulators associated with autophagy. It enters the nucleus and binds to the promoters of autophagy-related genes, enhancing their expression and promoting autophagy [118–120]. However, this study found that the role of FOXO3 in autophagy regulation was contrary to general studies, and the molecular mechanism behind this discrepancy remains unclear, necessitating further investigation.

### m6A modification and autophagy in lung cancer

In the context of small cell lung cancer (SCLC) (Fig. 4), the presence of m6A modification is closely linked to chemoresistance. SCLC is known for its highly aggressive nature, and although the combination of cisplatin and etoposide chemotherapy is initially effective for most patients, a significant number of patients eventually develop chemoresistance, resulting in rapid tumor progression. Research studies have indicated that METTL3 is highly expressed in chemotherapy-resistant SCLC cell lines. This high expression of METTL3 is associated with a poor prognosis for patients. Furthermore, both in vivo and in vitro experiments have demonstrated that overexpression of METTL3 promotes chemoresistance in SCLC. Mechanistic investigations have revealed that METTL3 induces m6A methylation of Decapping Protein 2 (DCP2), leading to the degradation of DCP2. This degradation, in turn, triggers mitochondrial autophagy through the Pink1-Parkin pathway, ultimately resulting in chemoresistance in SCLC [121]. In non-small cell lung cancer (NSCLC), METTL3 is expressed at higher levels than in paired normal tissues and is involved in gefitinib resistance. It does this by increasing the expression of the key genes of the autophagy pathway, ATG5 and ATG7, thereby positively regulating autophagy [112]. Another protein, IGF2BP2, is also highly expressed in NSCLC and promotes the stabilization of lncRNA MALAT1 by a m6A-dependent mechanism. This, in turn, promotes the expression of the downstream target gene ATG12 and enhances autophagy and NSCLC proliferation [122]. However, there are studies that suggest that m6A modification promotes autophagy and inhibits NSCLC progression. For instance, FU et al. found that FTO, which is highly expressed in NSCLC, inhibits GAS5 expression and autophagy by decreasing lncRNA GAS5 m6A methylation levels. Inhibition of FTO facilitated autophagic death of NSCLC cells and inhibited tumor growth via the GAS5/UPF1/BRD4 pathway [123]. Similarly, Guo et al. reported that upregulation of ALKBH5 promoted the expression of the ubiquitin conjugating enzyme UBE2C, which in turn reduced the expression of ATG3 and LC3, leading to autophagy inhibition and NSCLC progression [90].

### m6A modification and autophagy in gastrointestinal tumors

**Fig. 5:**
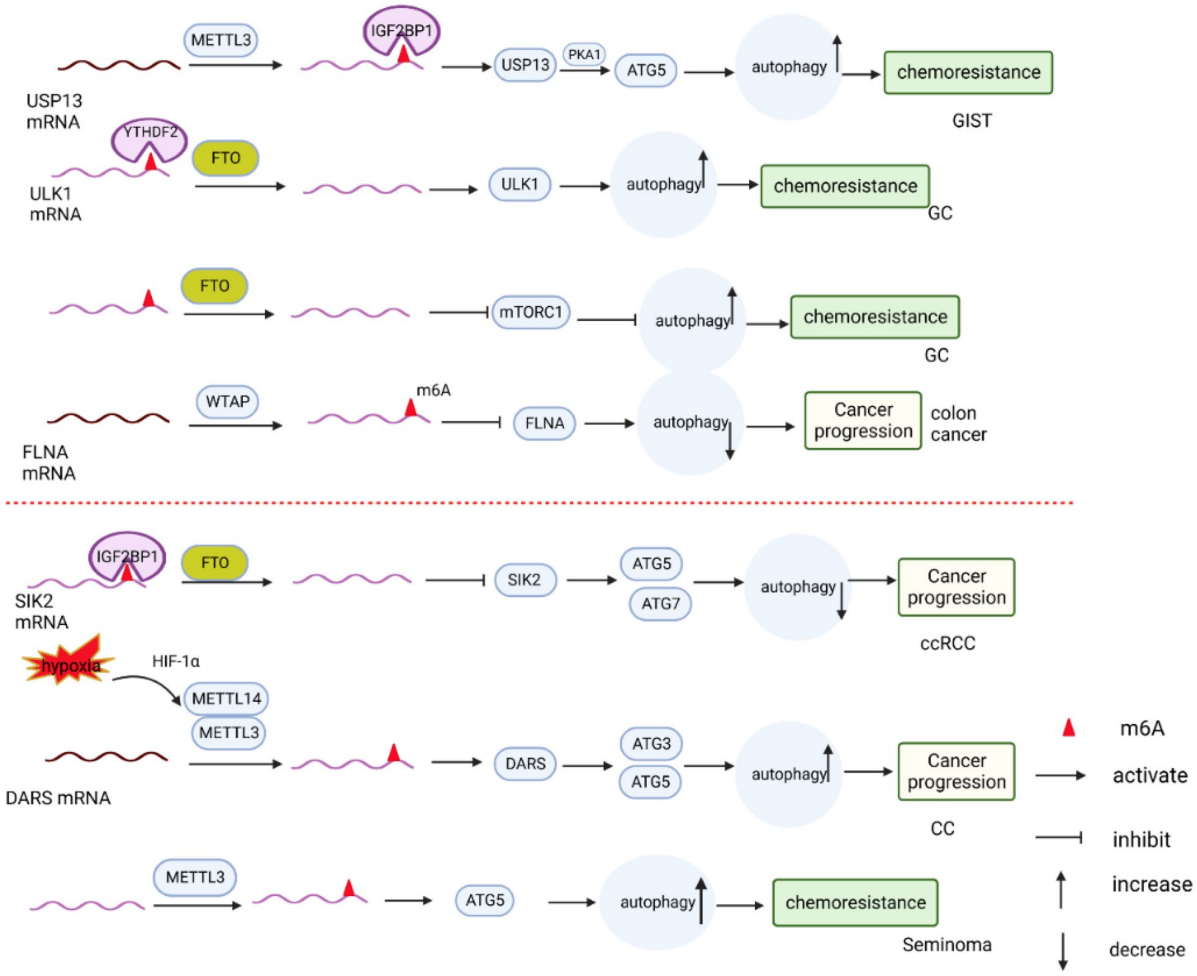
Role of m6A modification-mediated autophagy in gastrointestinal and genitourinary tumors, In gastrointestinal tumors, m6a modification impacts the expression of target genes such as USP13, ULK1, mTOR, FLNA, etc., leading to the regulation of autophagy levels and influencing the progression of gastrointestinal tumors. Similarly, in genitourinary tumors, m6a modification affects the expression of target genes such as SLK2, DRAS, ATG5, etc., thereby regulating autophagy levels and impacting the progression of genitourinary tumors

In the treatment of gastrointestinal mesenchymal stromal tumors (GISTs) (Fig. 5), resistance to imatinib poses a significant challenge. This resistance is caused by various mechanisms, including autophagy and m6A methylation. Recent studies have shown that the proteins METTL3, USP13, PAK1, and ATG5 are upregulated in drug-resistant tumors. Furthermore, the expression of these proteins is positively correlated and associated with a poor prognosis in GIST patients. Mechanistic investigations suggest that the stabilization of USP13 mRNA is facilitated by IGF2BP2 in a METTL3-mediated m6A-dependent manner. Additionally, USP13, an essential deubiquitinating enzyme, stabilizes ATG5 with the involvement of the PAK1 serine/threonine protein kinase. This stabilization enhances autophagy and contributes to the development of imatinib resistance in GIST cells [124]. In gastric cancer, FTO has been identified as a potential target for cisplatin resistance. Studies have shown that in cisplatin-resistant gastric cancer cells, the expression level of FTO is significantly increased, while the level of m6A in total RNA is significantly decreased. Additionally, the level of autophagy is significantly increased in these cells. Conversely, when FTO is deleted, the level of autophagy decreases, and cisplatin-resistant cells become more sensitive to cisplatin treatment. Mechanistically, FTO targets ULK1 to regulate autophagy and cisplatin resistance in a m6A-dependent manner. The deletion of FTO leads to an increase in m6A in ULK1 mRNA, which is then recognized and degraded by YTHDF2. This degradation negatively regulates autophagy and enhances the sensitivity of cisplatin-resistant cells to cisplatin [125]. Furthermore, FTO inhibits mTORC1, a negative regulator of autophagy, and promotes pro-survival autophagy, ultimately leading to chemoresistance in gastric cancer [126]. In colon cancer, WTAP exhibits high expression in colon cancer tissues and cells. It mediates the m6A modification of the tumor suppressor FLNA mRNA, resulting in the inhibition of FLNA expression. This inhibition suppresses autophagy and promotes colon cancer cell proliferation [90].

### m6A modification and autophagy in the genitourinary system

In clear cell renal cell carcinoma (ccRCC) (Fig. 5), the expression of FTO was found to be elevated, while m6A modification levels were reduced compared to neighboring noncancerous tissues. This increase in FTO expression was strongly associated with autophagy and poor patient prognosis. Additionally, when FTO was downregulated, it resulted in an increase in autophagic flux by targeting ATG5 and ATG7. This downregulation of FTO also inhibited the growth and metastasis of ccRCC both in vivo and in vitro. Mechanistically, the downregulation of FTO led to an increase in the level of m6A modification of SIK2 mRNA. This modified mRNA was then recognized by IGF2BP2 in a m6A-dependent manner, which increased its transcript stability and expression. SIK2 promoted the autophagy pathway, leading to the inhibition of ccRCC progression [127].

In cervical cancer (CC), HIF1α induces the upregulation of lncRNA DARS-AS1 under hypoxic conditions. This promotes protective autophagy and tumor cell survival. Mechanistically, DARS-AS1 recruits METTL3 and METTL14 to enhance the stability and translation of DARS mRNA, a cytoplasmic aspartic acid-tRNA synthetase gene, in a m6A-dependent manner. This subsequently increases the expression of ATG5 and ATG3, which affects autophagy in CC cells. This mechanism helps tumor cells adapt to hypoxic conditions and promotes their survival [128]. Moreover, RBM15 expression is significantly elevated in HPV-positive cervical cancer cell lines. RBM15 binds to c-myc mRNA, leading to an increase in the m6A level and protein expression of c-myc. This downregulates autophagy and promotes cervical cancer cell growth [129].

In seminomas, the majority of patients exhibit high sensitivity to cisplatin. However, some patients experience poor treatment outcomes due to cisplatin resistance [130, 131]. Research has shown that the expression of METTL3 is significantly higher in cisplatin-resistant TCam-2/CDDP cell lines than in cisplatin-sensitive TCam-2 cell lines. Moreover, when METTL3 was overexpressed in TCam-2 cells, the proliferation rate in the presence of cisplatin was significantly higher than that in the METTL3 knockdown group. This suggests that overexpression of METTL3 enhances the cisplatin resistance of TCam-2 cells. Mechanistic studies indicate that METTL3 directly targets the ATG5 transcript and promotes ATG5 expression in a m6A-dependent manner. This promotes autophagy and resistance to spermatogonial tumors.

### m6A modification and autophagy in head and neck tumors

**Fig. 6:**
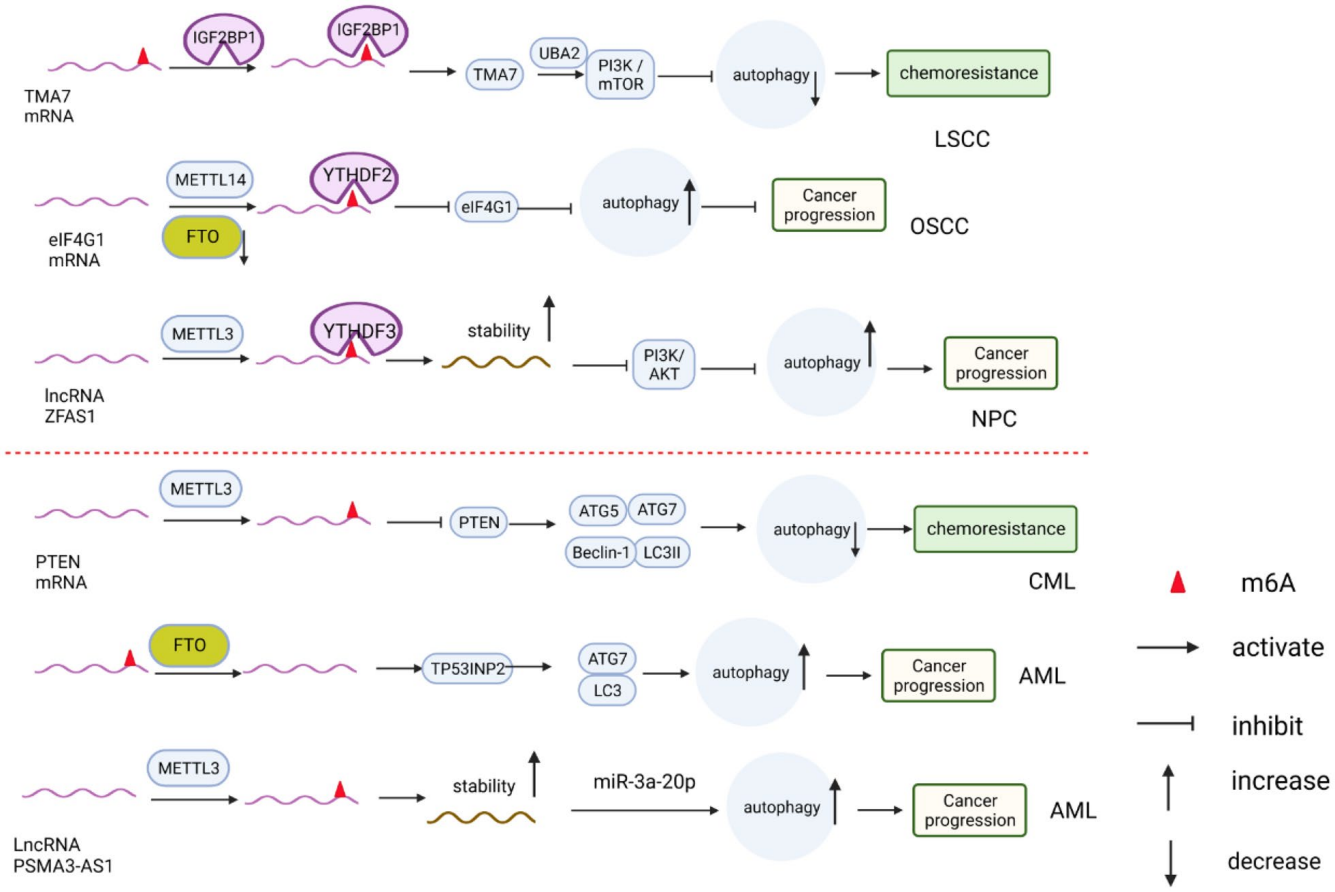
Role of m6A modification-mediated autophagy in head and neck tumors and leukemia, In head and neck tumours, m6a modification impacts the expression of target genes like TMA7, eIF4G1, and the stability of LncRNA ZFAS1. This modification also regulates autophagy levels and influences the progression of head and neck tumours as well as chemotherapy resistance. Similarly, in leukaemia, m6a modification affects the expression of target genes such as PTEN, TP53INP2, and the stability of LncRNA PSMA3-AS1. This modification also regulates the level of autophagy and affects the progression of leukaemia, particularly in terms of chemotherapy resistance

In laryngeal squamous cell carcinoma (LSCC) (Fig. 6), the upregulation of IGF2BP3 and TMA7 in LSCC tissues reduces autophagy levels and promotes LSCC progression and cisplatin resistance. Mechanistically, the m6A-methylated reader IGF2BP3 enhances the stability of TMA7 in a m6A-dependent manner. TMA7 interacts with UBA2 to inhibit autophagy in LSCC through the PI3K/mTOR signaling pathway, thus promoting the proliferation, invasion, migration, and colony-forming ability of LSCC cells and inducing resistance to cisplatin [132]. In oral squamous cell carcinoma (OSCC), FTO expression is elevated and correlates with autophagic flux. Knockdown of FTO expression in OSCC cell lines enhances autophagic flux and suppresses malignant tumor behavior. The underlying mechanism involves increased m6A modification of the target gene eIF4G1 mRNA after FTO silencing. YTHDF2 captures m6A-modified eIF4G1 mRNA, leading to its degradation. This results in reduced expression of the eIF4G1 protein, a negative regulator of autophagy, which promotes autophagy and inhibits tumor malignant behavior [133]. Furthermore, overexpression of METTL14 increases the m6A modification level of eIF4G1 mRNA, inhibiting its expression, enhancing autophagy, and suppressing migration, invasion, and proliferation of OSCC [114]. In the context of nasopharyngeal carcinoma (NPC), it was observed that the levels of m6A modification and METTL3 expression were significantly higher in tumor tissues than in neighboring tissues. Mechanistic studies revealed that METTL3 increased the m6A level of the long noncoding RNA ZFAS1. This, in turn, enhanced the stability and expression of ZFAS1 through the involvement of the reader protein YTHDF3. Furthermore, ZFAS1 competitively bound to miR-100-3p, leading to the promotion of ATG10 expression by inhibiting the PI3K/AKT pathway, which promoted autophagy processes and NPC cell proliferation, migration and tumor growth [90].

### m6A modification and autophagy in leukemia

In chronic myelogenous leukemia (CML) (Fig. 6), METTL3 decreases the expression of PTEN by increasing the m6A modification level of PTEN mRNA. This leads to downregulation of the autophagy-related proteins LC-II, Beclin-1, ATG5, and ATG3, inhibiting autophagy and promoting chemical resistance in CML [134]. In acute myeloid leukemia (AML), TP53INP2 is upregulated by FTO-mediated modification of m6A, which enhances autophagy activity by promoting the interaction of LC3 and ATG7, ultimately promoting leukemia cell survival [135]. Additionally, METTL3 enhances the stability of the lncRNA PSMA3-AS1, which further regulates ATG16L1 by targeting miR-3a-20p. This modulation of autophagy levels contributes to the progression of AML.

## Tumor control strategies based on m6A modification and autophagy

New therapeutic targets in oncology research are highly exciting. Numerous studies have shown that aberrant autophagy promotes tumor progression and chemoresistance. Autophagy provides energy for tumor cells to adapt to adverse environments, leading to tumor progression and drug resistance. Recent findings indicate that m6A modifications and their regulators play a crucial role in autophagy-mediated tumor progression and anticancer drug resistance. Therefore, targeting m6A modifications to counter autophagy-associated tumor progression and drug resistance shows promise as a therapeutic strategy. For instance, in a mouse model of hepatocellular carcinoma, knockdown of YTHDF1 significantly inhibited autophagy and tumor growth [115]. Similarly, in OSCC, downregulation of FTO and overexpression of METTL14 enhanced autophagy while inhibiting cell proliferation, migration, invasion, and tumor growth [114, 133]. In ccRCC, downregulation of FTO enhanced autophagic flux, leading to inhibition of tumor growth and metastasis in vivo and in vitro. Moreover, the small molecule inhibitor FB23-2, which targets FTO, suppressed tumor growth in a ccRCC mouse model, indicating that FTO is a potential druggable target and that FTO inhibitors hold therapeutic potential for tumor treatment [127].

Tumor drug resistance is a significant challenge in tumor therapy. Autophagy, in most tumors, plays a role in providing the energy required to evade radiotherapy-induced apoptosis, thus promoting tumor cell survival and leading to treatment resistance. Consequently, autophagy has been proposed as a cytoprotective mechanism contributing to the generation of drug resistance. Inhibiting autophagy could potentially be a strategy to reverse drug resistance in various tumors. For instance, in hepatocellular carcinoma, METTL3 stabilizes FOXO3 in a m6A-dependent manner, inhibiting autophagy and increasing the sensitivity of HCC to sorafenib [117]. Inhibition of mitochondrial autophagy by the METTL3 inhibitor STM2457 in SCLC has been shown to reverse chemoresistance in both in vivo and in vitro models [121]. In NSCLC, β-elemen reduces m6A methylation levels in gefitinib-resistant cells, inhibiting the cellular autophagy process and reversing gefitinib resistance [112]. In gastrointestinal mesenchymal tumors, inhibitors of USP13 attenuate ATG5 and inhibit m6A modification-mediated autophagy. Coadministration with 3-methyladenine enhances the therapeutic effect of imatinib in tumor model mice [124]. FTO knockdown in cisplatin-resistant gastric cancer cells (SGC-7901/DDP) inhibits ULK1-mediated autophagy and reverses cisplatin resistance both in vivo and in vitro [125]. Furthermore, targeted inhibition of m6A modification-mediated autophagy increases tumor sensitivity to cisplatin in various tumors, including laryngeal squamous cell carcinoma and seminomas [132, 136].

Autophagy, regulated by m6A modification, plays a crucial role in tumor progression. Targeting m6A regulatory factors can either promote or inhibit autophagy, offering a potential strategy to suppress tumor development and reverse drug resistance. Notably, autophagy tends to enhance chemotherapy resistance in tumors. Therefore, targeting m6A regulatory factors to inhibit autophagy could be an effective approach to overcome drug resistance. These studies open up new avenues for the prevention and treatment of tumors, as well as the reversal of chemotherapy resistance, by focusing on autophagy and m6A. However, further clinical studies are needed to fully explore the diagnostic, therapeutic, and prognostic value of m6A modification and autophagy in cancer.

## Conclusion and outlook

Both autophagy and m6A modification play dual roles in tumors. The role of m6A modification in cancer is reflected in its regulation of the expression of cancer-related genes. M6A modification is able to regulate multiple target genes and, depending on the “reader”, can either inhibit or promote the expression of the target genes. This duality of m6A modifications in tumors can be explained by their ability to act as either tumor-promoting factors or tumor-suppressing factors, depending on whether they promote or inhibit oncogenes (Fig. 3A). Autophagy, on the other hand, functions by removing misfolded proteins, damaged organelles, and ROS, providing cells with the energy needed for metabolism and exerting a protective effect. This protective role of autophagy can also be exploited by cancer cells, which utilize autophagy to obtain nutrients and energy in response to environmental stresses and unfavorable stimuli, leading to rapid tumor progression and therapeutic resistance. However, metabolic stress-induced autophagy can also inhibit necrosis, thereby limiting inflammatory responses and potentially inhibiting tumor growth [137]. Moreover, excessive progressive autophagy may be cytotoxic, causing cell death and potentially inducing tumor cell death as well. Autophagy has a dual impact on cells, exhibiting both protective and cytotoxic effects. These effects can also influence cancer cells, thereby contributing to the intricate and contradictory nature of autophagy in cancer. The precise role of autophagy in tumors is contingent upon various factors including the level of autophagy, intra- and extracellular environments, tumor type, stage, and genetic background. M6A modification plays a dual role in the regulation of autophagy. It can directly affect the expression of ATG genes or autophagy regulators to regulate autophagy. When the target gene of m6A modification is an ATG gene, it promotes autophagy by enhancing the expression of the ATG gene through positive 'reader' action. Conversely, it inhibits autophagy by reducing the expression of the ATG gene through negative 'reader' action. Additionally, m6A modification can regulate autophagy by influencing the regulators of autophagy. It promotes autophagy by enhancing the positive regulators or suppressing the expression of negative regulators. Conversely, it inhibits autophagy by suppressing the positive regulators or promoting the expression of negative regulators. For example, changes in METTL3 expression can inhibit and promote autophagy by affecting FOXO3 and DCP2, respectively [117, 121]. In conclusion, the regulation of autophagy in cancer through m6A modification depends on its impact on the expression of target genes and the reciprocal effect of these target genes on autophagy regulation. The precise role of m6A modification-mediated autophagy in cancer is determined by its influence on the level of autophagy and the specific characteristics of the cancer under investigation (Fig. 3B).

Due to the significant role of m6A methylation modification and autophagy in tumor progression, modulating m6A modification and autophagy can be an effective strategy for the prevention and treatment of cancer. Recent studies have highlighted the importance of m6A modification-mediated autophagy in cancer progression, particularly in drug resistance. By intervening in m6A modification or autophagy, it may be possible to halt cancer progression and enhance the sensitivity of chemotherapeutic agents. These current studies have demonstrated the tremendous therapeutic potential of targeting m6A modification and autophagy, which holds great research value and a promising future. However, due to their dual roles in cancer, simply inhibiting or enhancing autophagy and m6A modification will not lead to successful cancer treatment. The unclear roles of these processes in cancer raise concerns about their clinical value as therapeutic targets. Therefore, understanding the mechanism by which m6A modification regulates the dynamic balance of autophagy is crucial. As our understanding of the pathogenesis deepens, it will contribute to the development of effective clinical treatments for cancer. However, our current knowledge in this emerging field is still limited, and further studies are needed to expand our understanding in this area.

## Data Availability

Not applicable.

## Abbreviations

ATG: Autophagy-related proteins
m6A: N6-methyladenosine
mTORC1: Rapamycin complex 1
ULK1: Unc-51-like kinase
FIP200: Focal adhesion kinase family interacting protein of 200 kDa
PI3K: Phosphatidylinositol 3-kinase
LC3: Microtubule-associated protein light chain 3
mTOR: Mammalian target of rapamycin
METTL3: Methyltransferase-like 3
METTL14: Methyltransferase-like 14
WTAP: Wilms’ tumor 1-associating protein
METTL16: Methyltransferase-like 16
RBM15: NA binding motif protein 15
FTO: Fat mass and obesity associated
ALKBH5: Alpha-ketoglutarate-dependent dioxygenase alkB homolog 5
YTH: YT521-B homology
YTHDC1-2: YTH domain-containing proteins 1–2
THDF1-3: TH domain-containing family proteins 1–3
IGF2BPs: Insulin-like growth factor 2 mRNA-binding proteins
AVEGFA: Vascular endothelial growth factor A
NSCLC: Non-small cell lung cancer
HCC: Hepatocellular carcinoma
SCLC: Small cell lung cancer
DCP2: Decapping Protein 2
GISTs: Gastrointestinal mesenchymal stromal tumors
ccRCC: Clear cell renal cell carcinoma
CC: Cervical cancer
LSCC: Laryngeal squamous cell carcinoma
OSCC: Oral squamous cell carcinoma
NPC: Nasopharyngeal carcinoma
CML: Chronic myelogenous leukemia
AML: Acute myeloid leukemia
